# Urolithiasis Symptoms and Risk Factors Among the General Population of Alahsa, Saudi Arabia

**DOI:** 10.7759/cureus.39645

**Published:** 2023-05-29

**Authors:** Abdullatif K Almaghlouth, Hatim M Alqutayfi, Abdullah H Bohamad, Abdulhakeem J Almarzooq, Mohammed A Alamer, Danah J Alqattan

**Affiliations:** 1 Urology, King Faisal University, Hofuf, SAU; 2 Internal Medicine, King Faisal University, Hofuf, SAU

**Keywords:** awareness, alahsa, saudi arabia, urolithiasis, renal stone

## Abstract

Objective: This study aims to assess the awareness and attitudes about urolithiasis among the general population of Alahsa, Saudi Arabia.

Methodology: A cross-sectional study was conducted in September 2022 in Alahsa, Saudi Arabia, using a validated questionnaire that was distributed to the general population. Inclusion criteria include male and female citizens of Saudi Arabia who were living in Alahsa, older than 18 years old, and willing to participate in the study. Exclusion criteria include non-Saudi citizens or Saudi citizens who did not live in Alahsa. Data were analyzed using SPSS Statistics.

Result: The results showed there were 1023 participants. The results showed the awareness level of symptoms associated with kidney stones at 29%, complications at 34%, diagnosis at 51%, and treatment at 16%. The results showed there was a significant association between a history of kidney stones and having no complications (p-value = 0.002) or inflammation (p-value = 0.009). However, there was no significant association between kidney stone symptoms and the participants' comorbidities.

Conclusion: According to our findings, there was a low level of knowledge about the condition and the methods to prevent it, like dietary and lifestyle modifications. Regardless of the low level of general knowledge, there was some awareness of urolithiasis in some elements. Therefore, it is advised to increase health awareness campaigns.

## Introduction

The term "urolithiasis" refers to the formation of stones or calculi in the urinary tract, mostly in the kidneys and ureters, although it may also affect the bladder or urethra. There are many classifications according to their chemical composition, such as calcium, cystine, struvite, uric acid, and other rare types of stones [1].

It is one of the most common urological conditions, and its incidence is rising worldwide. It represents a significant health problem affecting diverse age groups, with a high prevalence in Saudi Arabia and a lifetime risk of 20%. It is more common among men than women, with a ratio of (3.2:1) [2]. The high incidence and prevalence can be linked to the hot climate of Saudi Arabia, which led to an increase in perspiration, which will lead to dehydration, as well as some local dietary habits, like decreased fluid intake, increased intake of animal proteins, high salt intake, decreased calcium intake, and genetic factors increasing crystallization and stone formation [3-5]. Urolithiasis patients are usually asymptomatic. Some patients, however, may experience loin pain, dysuria, hematuria, sweating, pallor, vomiting, restlessness, and urinary tract infection or obstruction [6]. Nowadays, with conservative management, most stones pass, and the rest are managed with minimal surgical interventions [7].

A study conducted in Malaysia showed that there are low levels of awareness about risk factors and symptoms of renal stones, and there is a lack of sufficient knowledge about the role of diet in the medical management of renal stones [8]. Another study conducted in Saudi Arabia showed the level of awareness about risk factors and prevention among communities. However, it seems that few Saudis, particularly those outside the medical profession, are familiar with these risk factors [9]. This raises the importance of awareness of urolithiasis to prevent such complications. Our study aims to assess the awareness and attitudes toward urolithiasis symptoms and the proper diet for this disease among the general population of Alahsa, Saudi Arabia.

## Materials and methods

Study design and selection criteria

A cross-sectional study was conducted in Alahsa, Saudi Arabia, using a validated questionnaire. Inclusion criteria include male and female Saudi Arabian citizens living in Alahsa, older than 18 years old, and willing to participate in the study. Exclusion criteria include non-Saudi citizens or Saudi citizens who did not live in Alahsa and who had refused to participate or had not completed the entire questionnaire.

Questionnaire

The questionnaire was distributed via social media networks including WhatsApp, Telegram, and Twitter to the general population. It was distributed between November 2022 and December 2022 in Arabic via social media networks including WhatsApp, Telegram, and Twitter. The questionnaire might take approximately four minutes to be completed. The questionnaire was divided into three sections. The first section included informed consent and demographic data such as age, gender, marital status, educational level, and occupational status. The second section included questions regarding the awareness of the prevalence of urolithiasis. The final section included questions that assess one's attitude toward urolithiasis. The knowledge and awareness level was calculated by giving the correct answer a score of 1 and the wrong answer a score of 0.

Ethical consideration

The participants’ confidentiality and the privacy of their data were the priorities. Nothing leads to any ethical issue being used, such as the names of participants or consent obtained through a direct question before completing the questionnaire. The approval was obtained from the Ethics Committee of King Faisal University. The code of approval is (KFU-REC-2022-OCT-ETHICS225).

Data management and statistical analysis

Data were analyzed using SPSS Statistics version 22.0 (IBM Corp. Released 2013. IBM SPSS Statistics for Windows, Version 22.0. Armonk, NY: IBM Corp.). The means and standard deviations used to describe the quantitative data frequencies and percentages (%) were used to describe categorical variables. The chi-square test was used to assess the association.

## Results

There were 1023 participants: 37.4% were between the ages of 18 and 24, 21.4% were between the ages of 25 and 35, 30.4% were between the ages of 35 and 50, and 10.8% were older than 50; 48% were male and 52% were female; 0.9% had primary education, 2.4% had middle education, 25.7% had secondary education, 15.6% had a diploma, and 55.3% had a bachelor's or postgraduate degree; 36.5% were single, 61.2% were 61.2% were married, 2% were divorced, and 0.4% were widowed (Table 1).

**Table 1:** Demographic data

Variables	Categories	N	%
Age	18-24 years old	383	37.4%
	25-35 years old	219	21.4%
	35-50 years old	311	30.4%
	More than 50 years old	110	10.8%
Gender	Male	491	48.0%
	Female	532	52.0%
Education level	Primary	9	0.9%
	Middle education	25	2.4%
	Secondary	263	25.7%
	Diploma	160	15.6%
	Bachelor and postgraduate	566	55.3%
Marital status	Single	373	36.5%
	Married	626	61.2%
	Divorced	20	2.0%
	Widowed	4	0.4%

The findings revealed a link between demographic data (age, gender, educational level, and marital status) and kidney stones, with a p-value <0.05 (Table 2).

**Table 2:** Association between demographic data and kidney stone

		Have you had kidney stones before?							
		Yes, I was diagnosed by a doctor		Yes, but I wasn't diagnosed by a doctor		No			
		N	%	N	%	N	%	Chi-square	p-value
Age	18-24 years old	10	1.0%	5	0.5%	368	36.0%	85.624	0.00
	25-35 years old	10	1.0%	0	0.0%	209	20.4%		
	35-50 years old	49	4.8%	6	0.6%	256	25.0%		
	More than 50 years old	30	2.9%	2	0.2%	78	7.6%		
Gender	Male	63	6.2%	5	0.5%	423	41.3%	11.068	0.004
	Female	36	3.5%	8	0.8%	488	47.7%		
Education level	Primary	1	0.1%	0	0.0%	8	0.8%	16.263	0.039
	Middle education	5	0.5%	1	0.1%	19	1.9%		
	Secondary	23	2.2%	3	0.3%	237	23.2%		
	Diploma	26	2.5%	3	0.3%	131	12.8%		
	Bachelor and postgraduate	44	4.3%	6	0.6%	516	50.4%		
Marital status	Single	12	1.2%	4	0.4%	357	34.9%	29.952	0.000
	Married	85	8.3%	9	0.9%	532	52.0%		
	Divorced	2	0.2%	0	0.0%	18	1.8%		
	Widowed	0	0.0%	0	0.0%	4	0.4%		

The findings revealed that 33% had a chronic disease, with anemia (12.6%), hypertension (8.9%), diabetes (5.4%), and asthma (1.9%) being the most common; the least common was lupus erythematosus, hepatitis C, osteoporosis, hydrocephalus, and myasthenia gravis (Table 3).

**Table 3:** Chronic diseases

Chronic disease	N	%
Anemia	140	12.6%
Hypertension	99	8.9%
Diabetes	60	5.4%
Asthma	21	1.9%
Hepatitis C	1	0.1%
Triglycerides	12	1.1%
Heart disease	7	0.6%
Crohn's disease	3	0.3%
Lupus erythematosus	1	0.1%
Thyroid disease	3	0.3%
Osteoporosis	1	0.1%
Irritable bowel syndrome	7	0.6%
Psoriasis	3	0.3%
Hydrocephalus	1	0.1%
Epilepsy	3	0.3%
Rheumatoid	3	0.3%
Myasthenia gravis	1	0.1%
No chronic disease	742	67.0%

The results showed there was a significant association between a history of kidney stones and side and back pain (p-value = 0.026), sweating (p-value = 0.002), and chills (p-value = 0.027), but there was no significant association with red or brown urine with blood, pain or burning during urination, nausea, fever, vomiting, and urine retention (Table 4).

**Table 4:** Symptoms that may be associated with kidney stones

		Have you had kidney stones before?							
		Yes, I was diagnosed by a doctor		Yes, but I wasn't diagnosed by a doctor		No			
		N	%	N	%	N	%	Chi-square	p-value
Side and back pain	No	10	1.00%	2	0.20%	195	19.10%	7.261	0.026
	Yes	89	8.70%	11	1.10%	716	70.00%		
Red or brown urine with blood	No	51	5.00%	6	0.60%	483	47.20%	0.313	0.855
	Yes	48	4.70%	7	0.70%	428	41.80%		
Pain or burning during urination	No	27	2.60%	4	0.40%	314	30.70%	2.120	0.347
	Yes	72	7.00%	9	0.90%	597	58.40%		
Nausea	No	72	7.00%	9	0.90%	715	69.90%	2.276	0.320
	Yes	27	2.60%	4	0.40%	196	19.20%		
Fever	No	89	8.70%	10	1.00%	784	76.60%	2.098	0.350
	Yes	10	1.00%	3	0.30%	127	12.40%		
Vomiting	No	78	7.60%	11	1.10%	790	77.20%	4.661	0.097
	Yes	21	2.10%	2	0.20%	121	11.80%		
Sweating	No	77	7.50%	8	0.80%	791	77.30%	12.155	0.002
	Yes	22	2.20%	5	0.50%	120	11.70%		
Chills	No	85	8.30%	12	1.20%	850	83.10%	7.199	0.027
	Yes	14	1.40%	1	0.10%	61	6.00%		
Urine retention	No	99	9.70%	13	1.30%	910	89.00%	0.123	0.940
	Yes	0	0.00%	0	0.00%	1	0.10%		
I don't know	No	96	9.40%	12	1.20%	793	77.50%	8.595	0.014
	Yes	3	0.30%	1	0.10%	118	11.50%		

The results showed there was a significant association between kidney stone prevention and drinking fluids (p-value = 0.039). Red meat increases the risk of developing kidney stones (p-value 0.01), tea and coffee help in the formation of kidney stones (p-value 0.01), and boiled parsley water helps prevent kidney stones (p-value 0.01). If a family member had kidney stones, this increases your risk of developing kidney stones (p-value = 0.002), but there was no significant association between kidney stone formation and urinary tract infections, sedentary lifestyle, excess calcium and uric acid in the blood, endocrine diseases, or drinking adequate amounts of water as prevention of stone formation (Table 5).

**Table 5:** Awareness of risk factors and prevention

		Have you had kidney stones before?							
		Yes, I was diagnosed by a doctor		Yes, but I wasn't diagnosed by a doctor		No			
		N	%	N	%	N	%	Chi-square	p-value
Drinking fluids prevent kidney stones from forming	No	8	0.8%	0	0.00%	144	14.1%	6.511	0.039
	Yes	91	8.9%	13	1.30%	767	75.0%		
Urinary tract infections increase the risk of developing kidney stones	No	57	5.6%	10	1.00%	516	50.4%	2.166	0.339
	Yes	42	4.1%	3	0.30%	395	38.6%		
Red meat increases the risk of developing kidney stones	No	48	4.7%	8	0.80%	680	66.5%	30.979	0.00
	Yes	51	5.0%	5	0.50%	231	22.6%		
A sedentary lifestyle increases the risk of developing kidney stones	No	56	5.5%	8	0.80%	618	60.4%	5.261	0.072
	Yes	43	4.2%	5	0.50%	293	28.6%		
Excess calcium and uric acid in the blood increases the risk of developing kidney stones	No	51	5.0%	7	0.70%	535	52.3%	1.998	0.368
	Yes	48	4.7%	6	0.60%	376	36.8%		
Tea and coffee help in the formation of kidney stones	No	66	6.5%	9	0.90%	759	74.2%	17.754	0.000
	Yes	33	3.2%	4	0.40%	152	14.9%		
Boiled parsley water helps prevent kidney stones	No	33	3.2%	7	0.70%	526	51.4%	21.531	0.000
	Yes	66	6.5%	6	0.60%	385	37.6%		
Endocrine diseases increase the risk of developing kidney stones	No	87	8.5%	12	1.20%	815	79.7%	0.357	0.837
	Yes	12	1.2%	1	0.10%	96	9.4%		
If you have kidney stones in a family member, this increases your risk of developing kidney stones	No	71	6.9%	11	1.10%	777	76.0%	12.226	0.002
	Yes	28	2.7%	2	0.20%	134	13.1%		
Drinking adequate amounts of water prevents the formation of kidney stones	No	99	9.7%	13	1.30%	910	89.0%	0.123	0.940
	Yes	0	0.0%	0	0.00%	1	0.1%		
I don't know	No	99	9.7%	13	1.30%	908	88.8%	0.370	0.831
	Yes	0	0.0%	0	0.00%	3	0.3%		

The results showed there was a significant association between kidney stones and having no complications (p-value = 0.002) and inflammation (p-value = 0.009) but no significant association with urethral obstruction, renal failure, diabetes, weakness in urination, or heart disease (Table 6).

**Table 6:** Expected complications of kidney stones

		Have you had kidney stones before?							
		Yes, I was diagnosed by a doctor		Yes, but I wasn't diagnosed by a doctor		No			
		N	%	N	%	N	%	Chi-square	p-value
Urethral obstruction	No	37	3.6%	3	0.3%	254	24.8%	4.135	0.127
	Yes	62	6.1%	10	1.0%	657	64.2%		
Renal failure	No	27	2.6%	6	0.6%	342	33.4%	4.566	0.102
	Yes	72	7.0%	7	0.7%	569	55.6%		
Diabetes	No	94	9.2%	12	1.2%	871	85.1%	0.404	0.817
	Yes	5	0.5%	1	0.1%	40	3.9%		
Weakness in urination	No	99	9.7%	13	1.3%	910	89.0%	0.123	0.940
	Yes	0	0.0%	0	0.0%	1	0.1%		
Heart disease	No	94	9.2%	12	1.2%	843	82.4%	0.779	0.677
	Yes	5	0.5%	1	0.1%	68	6.6%		
No complication	No	94	9.2%	12	1.2%	901	88.1%	12.274	0.002
	Yes	5	0.5%	1	0.1%	10	1.0%		
Inflammations	No	98	9.6%	13	1.3%	911	89.1%	9.342	0.009
	Yes	1	0.1%	0	0.0%	0	0.0%		
I don't know	No	86	8.4%	12	1.2%	736	71.9%	3.207	0.201
	Yes	13	1.3%	1	0.1%	175	17.1%		

The results showed there was a significant association between kidney stones and X-ray examination as a tool for diagnosis (p-value 0.01) but no significant association with kidney biopsy, urinalysis, blood tests, and stool examination (Table 7).

**Table 7:** Tests that help diagnose kidney stones

		Have you had kidney stones before?							
		Yes, I was diagnosed by a doctor		Yes, but I wasn't diagnosed by a doctor		No			
		N	%	N	%	N	%	Chi-square	p-value
Kidney biopsy	No	92	9.0%	12	1.2%	804	78.6%	2.122	0.346
	Yes	7	0.7%	1	0.1%	107	10.5%		
X-ray examination	No	14	1.4%	2	0.2%	337	32.9%	22.762	0.00
	Yes	85	8.3%	11	1.1%	574	56.1%		
Urinalysis	No	35	3.4%	4	0.4%	324	31.7%	0.130	0.937
	Yes	64	6.3%	9	0.9%	587	57.4%		
Blood tests	No	80	7.8%	9	0.9%	692	67.6%	1.531	0.465
	Yes	19	1.9%	4	0.4%	219	21.4%		
Stool examination	No	96	9.4%	11	1.1%	836	81.7%	4.398	0.111
	Yes	3	0.3%	2	0.2%	75	7.3%		
I don't know	No	96	9.4%	13	1.3%	762	74.5%	14.832	0.001
	Yes	3	0.3%	0	0.0%	149	14.6%		

The results showed that surgical intervention, parsley water, diuretic drugs, drinking milk daily, drinking barley, using medical therapy, drinking more water, and laser Lithotripsy and ultrasound lithotripsy had no role in the treatment of kidney stones (Table 8).

**Table 8:** Ways to treat kidney stones

		Have you had kidney stones before?							
		Yes, I was diagnosed by a doctor		Yes, but I wasn't diagnosed by a doctor		No			
		N	%	N	%	N	%	Chi-square	p-value
Surgical intervention	No	44	4.3%	6	0.6%	397	38.8%	0.056	0.973
	Yes	55	5.4%	7	0.7%	513	50.2%		
Drinking parsley water	No	46	4.5%	5	0.5%	444	43.4%	0.718	0.698
	Yes	53	5.2%	8	0.8%	466	45.6%		
Diuretic drugs	No	48	4.7%	7	0.7%	502	49.1%	1.609	0.447
	Yes	51	5.0%	6	0.6%	408	39.9%		
Drinking milk daily	No	93	9.1%	11	1.1%	877	85.8%	5.797	0.055
	Yes	6	0.6%	2	0.2%	33	3.2%		
Drinking barley	No	99	9.7%	13	1.3%	907	88.7%	0.370	0.831
	Yes	0	0.0%	0	0.0%	3	0.3%		
Using medical therapy	No	99	9.7%	13	1.3%	904	88.5%	0.743	0.690
	Yes	0	0.0%	0	0.0%	6	0.6%		
Drinking more water	No	98	9.6%	13	1.3%	901	88.2%	0.131	0.937
	Yes	1	0.1%	0	0.0%	9	0.9%		
Laser lithotripsy	No	99	9.7%	13	1.3%	907	88.7%	0.370	0.831
	Yes	0	0.0%	0	0.0%	3	0.3%		
Ultrasound lithotripsy	No	99	9.7%	13	1.3%	906	88.6%	0.494	0.781
	Yes	0	0.0%	0	0.0%	4	0.4%		
I don't know	No	84	8.2%	9	0.9%	770	75.3%	2.323	0.313
	Yes	15	1.5%	4	0.4%	140	13.7%		

The results showed the awareness level of symptoms associated with kidney stones at 29%, complications at 34%, diagnosis at 51%, and treatment at 16% (Table 9).

**Table 9:** Awareness level of kidney stones

Variables	Mean	Std. deviation	Percentage
Symptoms	2.65	1.78	29%
Kidney stones etiology	3.22	1.79	27%
Complication	1.35	0.74	34%
Diagnosis	1.54	0.90	51%
Treatment	1.10	0.71	16%

The results showed there was a significant association between kidney stone formation and a family history of stones (p-value = 0.008), the most appropriate amount of water to be taken daily (p-value = 0.002), and attending awareness campaigns about kidney stones in public places such as complexes or parks or via the Internet (p-value = 0.01). However, there was no significant association between kidney stones and the type of kidney stones that were most common or the amount of water you drink daily (Table 10).

**Table 10:** Factors associated with kidney stones

		Have you had kidney stones before?							
		Yes, I was diagnosed by a doctor		Yes, but I wasn't diagnosed by a doctor		No			
		N	%	N	%	N	%	Chi-square	p-value
Has anyone in your family suffered from kidney stones before?	Yes	62	6.1%	7	0.7%	396	38.7%	13.710	0.008
	No	27	2.6%	4	0.4%	362	35.4%		
	I don't know	10	1.0%	2	0.2%	153	15.0%		
What type of kidney stones are most common?	Cystine stones	2	0.2%	0	0.0%	11	1.1%	3.592	0.892
	Calcium stones	20	2.0%	2	0.2%	207	20.2%		
	Uric acid stones	11	1.1%	1	0.1%	64	6.3%		
	Struvite stones	1	0.1%	0	0.0%	11	1.1%		
	I don't know	65	6.4%	10	1.0%	618	60.4%		
How much water do you drink daily?	Less than 4 cups	19	1.9%	4	0.4%	226	22.1%	6.031	0.644
	4-6 cups	32	3.1%	5	0.5%	313	30.6%		
	6-8 cups	27	2.6%	3	0.3%	195	19.1%		
	8-10 cups	17	1.7%	0	0.0%	126	12.3%		
	More than 10 cups	4	0.4%	1	0.1%	51	5.0%		
What is the most appropriate amount of water to be taken daily?	Less than 1 L	10	1.0%	1	0.1%	22	2.2%	24.278	0.002
	1-2 L	33	3.2%	8	0.8%	394	38.5%		
	2-3 L	45	4.4%	2	0.2%	378	37.0%		
	More than 3L	9	0.9%	1	0.1%	81	7.9%		
	I don't know	2	0.2%	1	0.1%	36	3.5%		
Have you ever attended an awareness campaign about kidney stones in public places such as complexes or parks or via the Internet?	Yes	14	1.4%	0	0.0%	36	3.5%	20.621	0.000
	No	85	8.3%	13	1.3%	875	85.5%		

## Discussion

This study aims to investigate awareness and attitudes regarding urolithiasis symptoms among the general population of Alahsa, Saudi Arabia. The study assessed the awareness of 491 male (48%) and 532 female (52%) participants with a prevalence of 10% who were diagnosed with urolithiasis, the majority of whom were 49 (50%) among those 35-50 years of age, more commonly in males 63 (64%). There are studies that suggested a prevalence of 10-15% globally and up to 20% among Saudis. On the other hand, our study showed a 10% prevalence among the Alahsa population [8-10]. Our study found a higher incidence among those aged 35 to 50, which is consistent with many other studies [8,10,11].

The results showed that there was an increase in kidney stone incidences among males, making females less prone to develop kidney stones as has been widely reported in several studies [12-14].

Family history was significant for the individuals who were diagnosed with urolithiasis, with 63% of them having a relative who was also diagnosed; this association is seen among many other studies. Thus, it could be a genetic predisposition or due to the same dietary and lifestyle habits, but it was a significant point that should be taken into consideration since there are well-known familial causes of renal stones [11].

The results showed that some symptoms (nausea, vomiting, sweating, and chills) were poorly understood by the responders; however, flank pain, hematuria, and dysuria were better understood. A similar conclusion was reached in the study by Alghamdi et al. [3]. It could be due to a lack of knowledge or to the fact that these symptoms occur less frequently than flank pain, hematuria, and dysuria, which were found to be common in the study by Safdar et al. [11-12].

The findings revealed that the most identifiable risk factors associated with kidney stones were decreased fluid intake, red meat increased risk, and tea and coffee increased risk. While the two major factors promoting the development of urinary stones in the study by Baatiah et al. were low fluid consumption and daily caffeine consumption, and few participants mentioned obesity or family history as urolithiasis risk factors [15-17].

The results showed that the participants believed that X-rays were considered a tool for diagnosis while kidney biopsy, urinalysis, blood tests, and stool examination were not. It was in line with the study by Almuhanna et al. [9]. It is because several of the participants have a family member who has had a kidney stone.

The results showed that 29% of people were aware of the symptoms of kidney stones, 34% were aware of the complications, 51% were aware of the diagnosis, and 16% were aware of the treatment. Participants in the study by Baatiah et al. with similar findings had awareness levels that ranged from low (64.1%) to medium (35.3%) to high (0.6%) [17].

This study has some strengths in different aspects. The first strong point is the large sample size which reduces errors and biases. There is another point that makes the sample much better. There is a diversity of responses according to age, gender, and educational level. Accessibility to the questionnaire with the privacy of personal data makes the participants more open to participating. All of that ensures the external validity of the study result. The benefits of the study will be shown if it was conducted in a suitable area like in this study. Saudi Arabia has a high prevalence of urinary tract stones, and this is why it is important to make the community aware of the problem. This study was conducted in a limited region of Saudi Arabia, which is Alahsa. The exclusion criteria exclude non-Saudi Arabia citizens. The questionnaire focused on symptoms and risk factor awareness of kidney stones, and it did not include any questions regarding management awareness.

## Conclusions

We found that there was a low level of awareness of the disease and how to prevent it by making some alterations to lifestyle and dietary habits. Also, there was a strong association between the history of urolithiasis and age, gender, some chronic diseases, and some habits, like insufficient water intake, especially in a hot area like Saudi Arabia. This leads us to try to increase awareness by doing more studies and more health campaigns about urinary tract stones in Saudi Arabia. We recommend conducting a study with multiple regions with a larger sample size. The method of collection should be a personal interview rather than an online questionnaire.
